# Annotations

**Published:** 1898-07-30

**Authors:** 


### ANNOTATIONS.

### The Metropolitan Asylums Board.

The annual report of the Statistical Committee of the Metropolitan Asylums Board is always an interesting document, giving a record, as it does, of the year's working of one of the most important institutions for the control of infectious disease that our country possesses. This year the report is made additionally interesting from the fact that it contains, in its medical supplement, a series of papers by the medical officers of the various hospitals dealing with the cases which have come under their care. The inter-relations of scarlet fever and diphtheria are very carefully gone into, and it is shown that, during the year, 796 cases of post-scarlatinal diphtheria occurred out of a total of 15,241 completed cases of scarlet fever. It is difficult to gauge accurately the extent of the injury caused by diphtheritic infection in scarlet fever cases, but it is worthy of note that, putting on one side, of course, the convalescent hospitals, the hospital which has by far the lowest mortality among its scarlet fever cases is the one at which cases of diphtheria are not taken in. The number of cases of what are so spoken of in the report as "mistaken diagnosis" still continues to be very large, no fewer than 1,417 patients, or a percentage on the total admissions of 6·02, having been found after admission to the fever hospitals not to be suffering from the diseases mentioned in the medical certificates upon which they were removed. As in previous years, the largest number of such errors was found among the patients admitted to the Eastern Hospital, where the proportion was no less than 10·7 of the total. As regards scarlet fever the proportion of errors was 3·1 per cent., diphtheria cases 11·3 per cent., and enteric fever cases 44·1 per cent. Among the diseases which seem to have been mistaken for typhoid fever are: Pneumonia in 61 cases; phthisis, 11 cases; influenza 14 cases; and, curiously enough, constipation, 19 cases—the last-named error seeming to be almost a speciality of the North-Western Hospital, 16 of the cases having occurred there. In regard to diphtheria, the great difficulty seems to lie between it and tonsillitis; and, as to scarlet fever, tonsillitis, measles, and "no obvious disease" seem to be the chief stumbling-blocks.

### An Epidemic of Suspicion.

It is impossible to cast one's eyes over the newspapers of the day without becoming aware of the existence of an epidemic of suspicion in regard to medical men and all their ways. Every day we see great posters with sensational headings drawing attention to some new crime with which some doctor or other is being charged, and although we have urged, and continue to urge, that every possible step should be taken to root out from among us the abominable practices with which these charges have to do, we cannot but recognise that the condition of active suspicion into which the public mind has of late been thrown not only greatly complicates the daily work of medical men, but puts many estimable members of our profession in constant peril of false charges and consequent loss of reputation. What the public little knows about is the large part played in the married life of many women by premature expulsion of the ovum, and the large part played in general practice by what may be called "diseases of women," diseases which mostly require local examination to be made; and the point on which we would insist, and as to which we would urge upon all medical men the necessity of exercising the utmost caution for their own protection while the present epidemic of suspicion continues, is this, that whenever such an examination is made, however properly and in however good faith it may have been performed, if the woman should happen to miscarry within a week or so, the doctor is liable to be accused of most atrocious practices, the mere suggestion of which is enough to ruin his good name. The danger to which medical men are exposed in this respect in greatly enhanced by the fact that whether women who are *enceinte* be married or be single, they are equally unwilling to whisper a word about their condition until they are sure. Thus it happens that if women had their way, such investigations and examinations as are necessary would often be surrounded by an air of secrecy which could be turned with great effect against a medical man in case of a false charge being trumped up. We cannot, then, too strongly urge upon our professional brethren the necessity for caution in this matter. It would be well to take accurate notes of every examination, including records of the time, the surroundings, and the persons present or within call; and if it should be impossible to have a woman present, at least to have some servant "in and out" sufficiently frequently to do away with all appearance of secrecy. Among the upper classes there need be no difficulty—there is always the maid. Among the lower classes there is generally the district nurse or an old neighbour; but among the middle classes the doctor is apt to be cornered very uncomfortably; and in the present temper of the public, seeking for a scape-goat for its own vices, good repute will be found but a small protection. No pains can be too great for the preservation of one's good name.

### The Flash Point of Petroleum.

If the explanation of the Earl's Court explosion which was given by Captain Thomson and was accepted by the jury is correct, it furnishes another example of the dangers of a low flash petroleum and of the wisdom of the conclusion arrived at by the Petroleum Committee. It appears that the magazine contained gunpowder, bombs, petroleum, and sodium, the latter being contained in sealed tins. The custom was to open the tins outside the magazine, and then carry the pieces of sodium back and keep them immersed in a petroleum bath until they were required in the exhibition. The explanation given of the explosion is that a bit of the sodium must have dropped on the floor, and, rapidly absorbing oxygen, must have broken into a flame which, exploding the petroleum vapour arising from the bath, set fire to the oil and all the other contents of the magazine, including, of course, the gunpowder. If this is so, the conduction of the flame from the burning sodium to the oil was due to the explosive vapour which the latter gave off.

